# Dose-Book and Manual of Prescription Writing

**Published:** 1896-03

**Authors:** 


Dose-Book and Manual of Prescription Writing. With a list of Official Drugs and Preparations, and also many of the Newer Remedies now frequently used, with their doses. By E. Q. Thornton, M. D., Ph. G., Demonstrator of Therapeutics, Jefferson Medical College, Philadelphia; Acting Assistant Surgeon, United States Marine Hospital Service. 334 pages, printed on extra heavy paper. Price, cloth, $1.25. W. B. Saunders, Publisher, 925 Walnut St., Philadelphia.

This is one of Saunders' New Aid Series, and is one of the best and most practical books of the series. It will be found especially useful to the young physician just beginning his professional career, and a large per cent. of the older practitioners could study it profitably. The book treats of the composition and strength of all official preparations intended for internal administration, weights and measures, solubilities, incompatibilities; and space is given to the grammatical construction of prescriptions, accompanied by example prescriptions illustrating some of the methods of employing the different classes of remedies. The table of incompatibles is quite a large one, and is a very important part of the book. H.

